# BoneJ2 - refactoring established research software

**DOI:** 10.12688/wellcomeopenres.16619.2

**Published:** 2021-04-28

**Authors:** Richard Domander, Alessandro A Felder, Michael Doube

**Affiliations:** 1 Department of Comparative Biomedical Sciences, The Royal Veterinary College, University of London, London, NW1 0TU, UK; 2 Research Software Development Group, University College London, London, WC1E 7HB, UK; 3 Department of Infectious Diseases and Public Health, City University of Hong Kong, Kowloon, Hong Kong

**Keywords:** Open-source, Java, Bone, Biology, Morphometry, ImageJ, FIJI, BoneJ, Image analysis, Software engineering, Programming

## Abstract

Research software is often developed with expedience as a core development objective because experimental results, but not the software, are specified and resourced as a project output. While such code can help find answers to specific research questions, it may lack longevity and flexibility to make it reusable. We reimplemented BoneJ, our software for skeletal biology image analysis, to address design limitations that put it at risk of becoming unusable. We improved the quality of BoneJ code by following contemporary best programming practices. These include separation of concerns, dependency management, thorough testing, continuous integration and deployment, source code management, code reviews, issue and task ticketing, and user and developer documentation. The resulting BoneJ2 represents a generational shift in development technology and integrates with the ImageJ2 plugin ecosystem.

## Introduction

BoneJ ^1^ is a collection of bone image analysis plugins for the ImageJ ^2^ scientific image analysis software. A plugin is a piece of software that extends the capabilities of its parent application. For example, BoneJ provides a tool that counts connections within a structure by calculating an image’s Euler characteristic ( *χ*), a functionality that is absent from core ImageJ. Both ImageJ and BoneJ are free and open-source, which means that their source code is readily available online for anyone to inspect, download, use, modify and distribute, taking an open science approach by providing all methodological details. ImageJ plugins run on Windows, MacOS, GNU/Linux, and other operating systems because they are implemented in Java. Java programs are executed inside an operating system-agnostic Java Runtime Environment (JRE).

The plug-ins in BoneJ are intended for analysing computed tomographic (CT) and X-ray microtomography (XMT) images of whole bones, trabecular bone, and osteocyte lacunae. BoneJ was originally produced to satisfy a need to answer research questions using data to which extant software was ill-suited ^3– 5^. To enable other researchers to benefit from the development effort, plugins written by Doube and others were altered to suit the skeletal research domain (for example by using ASBMR standard histomorphometric nomenclature ^6^), bundled together in a single Java archive file ( BoneJ_.jar) for ease of installation, and basic user and developer documentation provided at bonej.org. BoneJ became popular for bone morphometry, and in other fields such as materials, soil, and food science. By 2020 its paper ^1^ was being cited in peer-reviewed literature 4–5 times per week ^i^.

In the time since BoneJ was released in 2010, the software ecosystem on which it relied made several advances. Schindelin and others implemented a software updater for ImageJ as part of their Fiji Is Just ImageJ ( Fiji) project ^7^, while ImageJ2 introduced a new *N*-dimensional image model and modern development practices such as modularity, dependency management ( Maven), continuous integration (Jenkins, then Travis, likely to be superseded by GitHub Actions), and community development and support protocols (GitHub, Gitter, image.sc forum) ^8^. At the same time the JRE version 6 that BoneJ was built for was phased out, the 3D libraries BoneJ’s plugins relied on (Java3D 1.5) were obsoleted, and the 3D Viewer ^9^ that BoneJ used for surface meshing and visualisation was abandoned. BoneJ was dependent on third-party software that was no longer maintained, while users found they could not install and run BoneJ in their environment creating a risk of forced obsolescence. 

At the same time, the developments in ImageJ2 and Fiji represented an opportunity to improve BoneJ’s engineering standard and user experience, as well as opening avenues for new algorithm development and implementation. ImageJ2’s new Ops framework and dependency management with Maven meant that libraries and plugins that had been copy-pasted into BoneJ_.jar, such as Analyze Skeleton ^10^, could be removed and used from a single location rather than duplicated, eliminating the overhead involved in maintaining copies of active projects. Maven combined with the ImageJ updater enabled updating resources such as the linear algebra library JAMA (last updated in 2012) to JOML (under active development) and to use Eclipse Collections’ fast IntHashSet implementation in place of the standard Java HashSet<Integer>.

Due to the increased complexity and engineering sophistication of the new ImageJ2 ecosystem, it became infeasible for BoneJ to remain a part-time, scientist-maintained project. This was especially so because as university faculty MD no longer had the long stretches of time needed to concentrate on software engineering, nor were software outputs or community support part of his employment performance criteria. In the UK, an awareness of *Research Software Engineers* (RSE) as valuable team members was growing ^11^. Here, we describe the steps taken to modernise the BoneJ project, and use BoneJ to provide a perspective on the role software engineering takes in the contemporary research ecosystem.

### Design

The top-level project identity has remained at bonej.org, which serves to direct users to project functions on third-party sites, rather than hosting content. The only content that remains in place at bonej.org is legacy documentation, for reproducibility purposes. Links at bonej.org direct users to user documentation at imagej.github.io, to the user forum at forum.image.sc, to developer resources including code and technical documentation at GitHub, and BoneJ’s social media account on Twitter. Project Java package declarations are now all in the form of org.bonej.package, to emphasise common ownership as distinct from the personal ownership implied by org.doube.bonej.package used in BoneJ1’s package declarations. Anonymous opt-in usage data are collated at Google Analytics, which helps determine which plugins are used most heavily, and which BoneJ, Java, and operating system versions are active in the user community. 

The starting point of designing BoneJ2’s codebase and development environment was to assess how the plugins in BoneJ1 needed to change to follow software development best practices, and to work with the new ImageJ platform. At the time (2016) BoneJ was one of the first projects attempting to transition to the new technology underlying ImageJ2. As pioneers we wanted not only to improve our own code, but also to provide an example that others could follow. After initial research into the design of BoneJ2, we devised a strategy for restructuring the software using wrapper plugins and proceeded to work on individual tools on a case-by-case basis.

**Figure 1.**
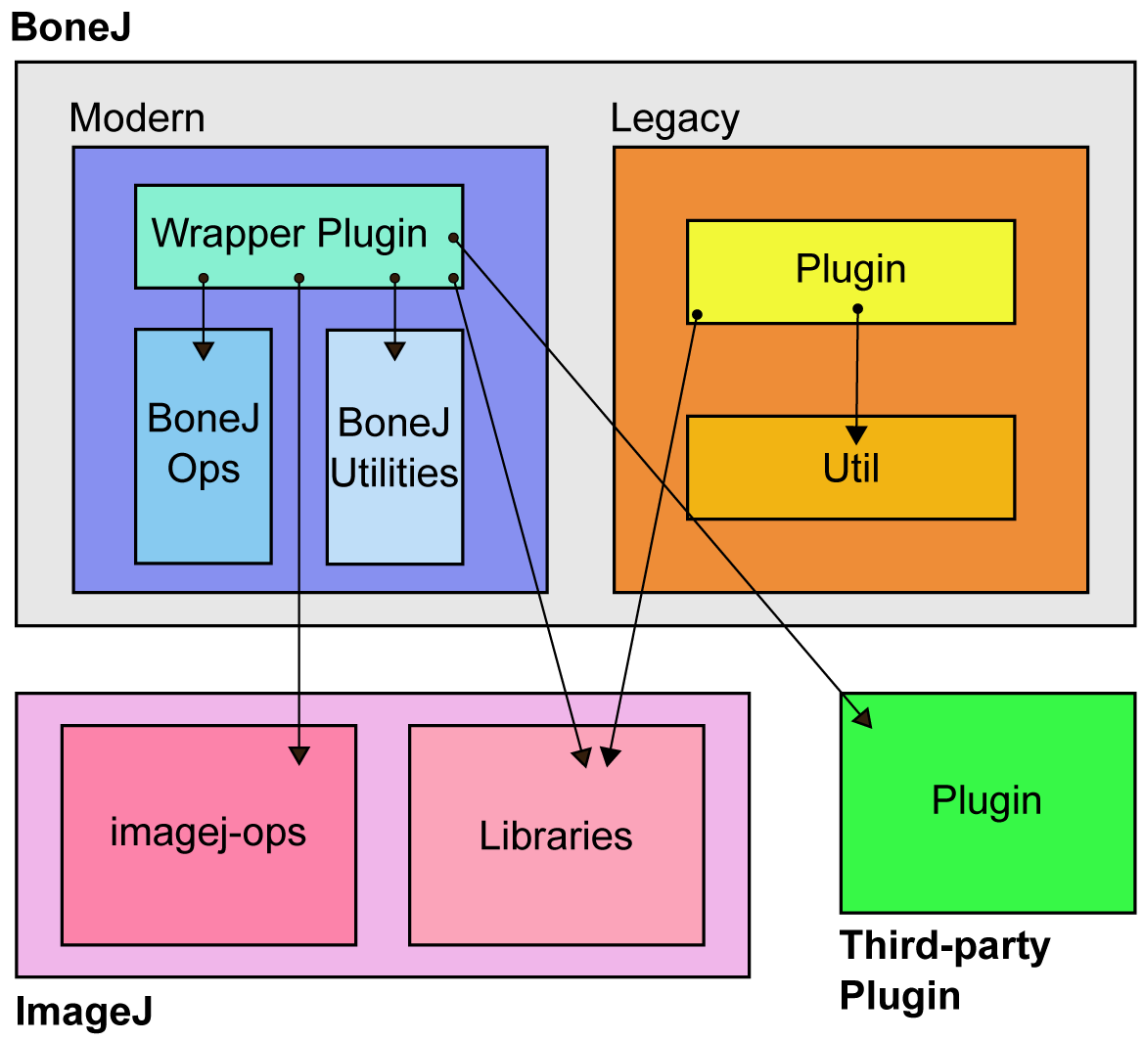
An overview of BoneJ architecture. Modern wrapper plugins call both ImageJ and third-party plugin code. They mainly run algorithms from the ImageJ Ops framework. Both Modern and Legacy plugins depend on various ImageJ libraries. ImageJ Ops and library architecture are described in more detail elsewhere ^8^. The presence of all the components necessary to run BoneJ is managed by Maven on the developer side and by the ImageJ Updater on the user side.

The wrapper plugins in BoneJ2 are intended to be light pieces of software that handle user interaction and call code from elsewhere. They orchestrate the multiple phases of execution that are needed to derive the results that users need to analyse their images ( Figure 1). Wrapper plugins also contain code specific to skeletal biology such as bone morphometry acronyms. Code common to all the wrappers is in a base class, BoneJCommand, which wrapper plugins access via Java *inheritance* to avoid code duplication. Using wrappers offers advantages such as reducing the amount of code to maintain and allowing rapid implementation of changes if a certain piece of software no longer serves our needs.

Wrapper plugins were designed to call code from external sources such as the ImageJ Ops framework and third party plugins. However, for some of the wrappers we needed to provide these "external" tools ourselves. We saw that we had to rewrite the majority of BoneJ1, and that some of the functionality there was generic and useful enough to become parts of ImageJ itself. We split the previously monolithic design into independent, reusable pieces, and made BoneJ2 a much lighter distribution. We wanted to further a design where the slight differences in functionality that different audiences need, such as domain-specific terms for measurements, could be added on top of a more basic, shared technology, enabling custom "remixes" of generic image analysis algorithms.

Ideally BoneJ2 would have only modern external dependencies. However, some necessary third-party tools had not been ported to the new technology, and it would be an unreasonable workload for us to reimplement them. Some third-party code will never be modernised because it is not maintained, or if it is maintained the maintainers may not want to modernise it in the near future. As a compromise we designed modern wrappers that encapsulate calls to legacy dependencies. Mixing modern and legacy code in ImageJ can introduce unwanted complexity so we limited them to as few carefully crafted code snippets as possible.

 In the early stages of BoneJ2’s project development it became apparent that it would take significantly longer to port BoneJ1 than initially anticipated. We intended to release BoneJ2 only once porting was complete but realised this would take too long to be able to then react to user feedback on the new version within the three year project time frame. We arranged the project into modules so that we could release the software incrementally, distributing a diminishing pool of legacy BoneJ1 plugins alongside a growing set of modernised tools.

 We released *experimental* versions of BoneJ2 in June 2017, where some of the plugins were new, but some were still exactly as they appeared in BoneJ1. With the name *experimental* we wanted to communicate that while some parts of BoneJ2 were ready to use, the software was still going to go through major changes. For example, we wanted to prepare users for the differences in the look-and-feel of the plugins as they changed from the legacy to modern implementation.

The ambition of moving code to core ImageJ created some challenges. We had to design the algorithms to suit not just our own but everybody’s anticipated needs. With this in mind, we tried to adapt the methods to suit as many kinds of images as possible. For example, previously the BoneJ1 plugin *Volume Fraction* handled only 3D images, whereas its Op can now process images with any number of dimensions. However, BoneJ2 includes only *Area Fraction* for 2D images and *Volume Fraction* for 3D images. With other plugins, such as *Connectivity*, we found that to support 2D images in addition to 3D images, we would have to develop a significantly different algorithm that would not fit within a single Euler characteristic-calculating Op. Only 3D Connectivity is included in BoneJ2 at present.

### Implementation

ImageJ and most of the related projects use *Git* ^12^ and *GitHub* for version management, and *Maven* for build automation, so those were natural choices for BoneJ2. Features that BoneJ uses have remained free of charge and unencumbered by third-party licence requirements. BoneJ adopts a standard naming scheme for its Git branches, with *master* containing release-ready code, with new features and bug fixes being developed on topic branches that are merged into *master* via pull requests.

BoneJ2 is a multi-module Maven project, meaning it consists of several JAVA archive (JAR) packages that reference each other like libraries. That is, they can access each other’s public application programming interfaces (APIs) only. Separating functionality into different modules during development makes it more difficult to unintentionally introduce tacit dependencies among modules’ code. The multi-module structure helps to organise code, and it makes functionality within BoneJ usable for others without them having to depend on the whole project. As mentioned in the *Design* section, BoneJ2 is split into *Legacy* and *Modern* parts. They are further divided into bonej-legacy-plugins_, bonej-legacy-util_, bonej-plugins, bonej-ops and bonej-utilities. The bonej-ops and bonej-utilities JARs contain code used by the wrapper plugins and could be thought of as the *skunkworks* of the project where code matures before it is ready to be engineered out of BoneJ and moved to parent projects. As more BoneJ code goes upstream into the core ImageJ platform, this level of structuring will become less necessary.

Table 1 details the status of all BoneJ1 plugins in relation to BoneJ2. The *wrapped external* plugins comprise new adaptor code that accesses third-party plugins that had been duplicated in BoneJ1 and subsequently removed from BoneJ2. They contain the small amount of custom functionality, where the BoneJ1 versions of the plugins differed from the originals. In some cases we also contributed to the original plugins. The benefits of not duplicating code are that there is a single upstream project to maintain, whose updates benefit BoneJ with no further downstream work. The secondary function of wrappers is to bundle the plugins into BoneJ, which makes sure they are available to other tools that need them, such as *Intertrabecular Angles* (ITA). The plugins marked *external* have no wrappers because they are included in ImageJ by default, and BoneJ1 added no functionality to them.

**Table 1.** Continuity table showing how plugins from BoneJ1 were carried forward.

Functionality	Status
Analyse Skeleton	wrapped external (Fiji plugin)
Anisotropy	ported
Connectivity ^♦^	ported
Delete Slice Range	legacy
Density Distribution ^‡^ *	ported external (PQCT)
Ellipsoid Factor	ported
Erode 3D, Dilate 3D	external (ImageJ plugins)
Fit Ellipsoid	ported
Fit Sphere	legacy
Fractal Dimension	ported
Help ^‡^	discontinued
Interpolate ROIs	ported external (ImageJ1)
Intertrabecular Angles	new
Isosurface ^★^	ported
ISQ Reader / Scanco ISQ	ported external (SCIFIO)
Kontron IMG	ported external (SCIFIO)
Moments of Inertia	legacy
Neck Shaft Angle	discontinued
Optimise Threshold	discontinued
Orientation ^‡^	legacy
Particle Analyser	legacy
Plateness	discontinued
Purify	legacy
Stratec pQCT	ported external (SCIFIO & PQCT)
Skeletonise 3D	wrapped external (Fiji plugin)
Slice Geometry	legacy
Structure Model Index	discontinued
Thickness	wrapped external (Fiji plugin)
Usage reporting ^♦^	ported
Volume Fraction ^†^	ported

★Renamed to *Surface area* †Split into *Surface fraction* and *Area/Volume fraction* ‡Added to BoneJ1 after the publication of Doube *et al*. (2010) ^1^ *Split into an independent plug-in ♦Both legacy and modern implementations are provided due to the higher performance of legacy code

*Ported* plugins are fully compatible with the modern ImageJ API and have no legacy dependencies. As much of their functionality as possible has been moved into the ImageJ Ops framework and other parts of the ImageJ platform. While ITA is the only entirely new tool, there are new features in all the ported tools. For example, *Connectivity* supports images with channels or time in addition to 3 spatial dimensions (hyperstacks), and *Anisotropy* uses a new method for sampling the image. After modernising the *ported external* plugins, we submitted them for inclusion in upstream or third-party projects where they fit more naturally. We added the plugins that read custom file formats to SCIFIO (SCientific Image Format Input and Output), so now ImageJ can open them without users needing to install BoneJ. The tools marked *legacy* are how they appeared in BoneJ1, but their dependencies are managed with Maven.

The pQCT plugins by Timo Rantalainen ^13^ were unbundled from BoneJ and placed in their own repository and with their own update site, because the BoneJ team was no longer needed to help with releases, and Rantalainen has always maintained control over the pQCT code and documentation. People who need only the pQCT tools can install them separately without BoneJ, and vice versa. The pQCT update site still includes a plugin for the custom *Stratec pQCT* file format even though it is also supported by SCIFIO. This is because SCIFIO is still experimental code, and we wanted to ensure full backwards compatibility.

*Discontinued* plugins were removed from BoneJ2 altogether. We removed the *Structure Model Index* (SMI) plugin because it does not measure rods and plates in the presence of the substantial concave curvature that is common in trabecular bone. Such curvature varies as a function of bone volume fraction (BV/TV) ^14^, which can lead to erroneous conclusions about changes in bone architecture. *Ellipsoid Factor* (EF) ^15, 16^ replaces BoneJ1’s SMI plugin and the early EF prototype *Plateness*. Due to the risk of misuse, and because it was doing little more for most users than ImageJ’s built-in auto-thresholder, we also elected to discontinue *Optimise Threshold*.

### Testing

During the code overhaul a major effort was made to improve the testing framework and coverage of BoneJ code. We added unit tests to cover more than 90% of the lines of BoneJ2 code. Unit tests execute code using standard conditions and check that the expected result is returned. A differing result indicates that something within the tested code, or code that it relies on, has changed and the developers are alerted by the continuous integration tools Maven and Travis. Code with failing unit tests cannot be merged into the *master* Git branch and thus is prevented from reaching the end user. BoneJ’s lower-level code has greater test coverage than higher level code because the unit tests are simpler to write and to include in the continuous integration framework. Testing low-level code thoroughly helps prevent bugs from appearing after implementing more complex software on top of it. BoneJ2 is also included in the SciJava project’s testing framework ^17^ to ensure that it uses a compatible build environment and compatible versions of dependencies and plugins and so that projects depending on BoneJ2 are also brought up to date. In this way BoneJ2 maintains tested version coordination with the rest of the large ImageJ/Fiji and wider inter-dependent scientific software ecosystem. User interaction testing is partly automated and partly manual. Ideally all tests would be automated, but if for example the code runs a UI call to show a dialog, there is no guarantee that the user will see the dialog. The manual tests are sets of use cases that define a set of repeatable steps of user interaction, and the expected behaviour of BoneJ in response. They are time-consuming, and thus usually performed only after major changes to a plugin. 

BoneJ1 plugins were validated against test images during their development, and continuity was ensured by checking that BoneJ2 results matched BoneJ1 results on the same test images. For some plugins results deviate because the technology has changed, for example, the marching cubes algorithm that produces meshes used to calculate surface area is implemented differently in ImageJ Ops than the legacy ImageJ 3D Viewer. As a result, the ratio reported by *volume fraction* differs by 0.6 % on the sample image bat-cochlea-volume.tif. Mean intercept vectors are generated differently in BoneJ2’s *Anisotropy* than in BoneJ1’s *Anisotropy*, and this introduces a systematic bias to degree of anisotropy calculations that is likely to vary depending on specifics of user images.

### Continuous integration and deployment

User builds of BoneJ2 are deployed after running all tests, updating the Maven artefact version numbers, Git tagging the commits and archiving a reference copy of the tagged code with a digital object identifier (DOI) at Zenodo (project doi:10.5281/zenodo.1427262 ^18^). The latest Maven artefacts, which are specific versions of the BoneJ JAR files, are uploaded to BoneJ’s ImageJ update site. Users are then reminded by the ImageJ updater to update their plugins. Plugin updates and their dependencies are listed by the updater, which retrieves them from the update site. Updated plugins become active after restarting ImageJ.

Since v7.0.0 ( *styloid*) BoneJ has been in a high-frequency release cycle, improving performance, fixing bugs, and adding small user features, and incrementing the patch version according to semantic versioning conventions. BoneJ2’s multi-module design means that for most of the minor and patch releases at least one of the modules contains no functional code changes. The semantic versioning relates to the BoneJ2 project as a whole and not to individual modules’ jar files, resulting in frequent ‘empty’ updates of jar files especially for the minor and patch revisions. Originally the release and versioning of each module was handled separately, but that proved too arduous in the long run.

### Operation

The minimal system requirements are:

- Windows, Mac OS X, GNU/Linux or other OS running Java 8 or later.
- ImageJ2 with the java-8 and with or without the Fiji update site enabled.
- Minimal hardware requirements are readily satisfied by a contemporary laptop computer, however, performance will scale with increasing CPU cores and RAM especially for operations running in multiple threads and on large data sets.

The tools in BoneJ are specialised and require a working knowledge of image processing. The purpose of developing BoneJ as an ImageJ plugin is to have ready access to its large ecosystem of filtering, segmentation, and thresholding tools for image preprocessing. Most BoneJ algorithms expect binary images, which have only two values: one for background and one for foreground. In BoneJ’s case the foreground is assumed to represent bone. It is the user’s responsibility to segment and threshold their images so that the foreground truly corresponds to bone, and not soft tissue, noise, or other imaging artefacts. The plug-ins do not and cannot determine if the binary images represent the samples adequately. Users are encouraged to run sensitivity analyses to determine how reasonable variations to their processing and analysis settings affect readout values. They should also check BoneJ’s output using test images, which could be synthetic images with known properties or images that represent well-characterised test objects with features relevant to the user’s research domain.

## Use cases

In this example, we calculate bone volume fraction (BV/TV), ellipsoid factor (Tb.EF), degree of anisotropy (DA), thickness (Tb.Th), separation (Tb.Sp), and connectivity density (Conn.D) of trabecular bone from the femoral head of *Apteryx haastii*, using the interactive menu-driven approach and an ImageJ macro. Variable parameters of all the plugins are documented at https://imagej.github.io/plugins/bonej. As a comparison, the same parameters were calculated with BoneJ 1.4.3, using ‘Auto Mode’ for *Anisotropy*

### Interactive use case

1. Install BoneJ according to the instructions at https://imagej.github.io/plugins/bonej#installation
2. Download umzc_378p_Apteryx_haastii_head.tif.bz2 from doi:10.6084/m9.figshare.7257179
3. Extract umzc_378p_Apteryx_haastii_head.tif from the .bz2 file. Note that it is a 16-bit greyscale X-ray microtomography image with isotropic pixel spacing (10.1 µm), and that the trabecular bone and bone marrow fill the entire image volume.
4. Run *Plugins > Macros > Record...* to capture the sequence of commands
5. Run *Process > Filters > Gaussian Blur (3D)* with a sigma of 2.0 in all 3 dimensions to smooth bone surfaces
6. Run *Image > Adjust > Threshold*, select Dark background and Stack histogram, click Auto and then click Apply
7. In the *Convert Stack to Binary* window, unselect all checkboxes and select *Method: Default, Background: Dark*, click OK
8. Pass a cursor over the image and check in the ImageJ status bar that the pixel value for trabeculae is 255 and marrow space 0. The inverting lookup table (LUT) indicates that foreground is displayed as black and background white.
9. Run *Plugins > BoneJ > Anisotropy* and select *Recommended minimums*, click OK to calculate DA
10. Run *Plugins > BoneJ > Fraction > Area/Volume Fraction* to calculate BV/TV
11. Run *Plugins > BoneJ > Ellipsoid Factor* with *Vectors:100, Sampling Increment: 0.435, Skeleton points per ellipsoid:10, Contact sensitivity:1, Maxmimum iterations: 50, Maximum drift:1.73, Minimum semi-axis: 1, Repetitions: 3, Average over largest n ellipsoids: 3, Seed points based on distance ridge: true, Threshold for distance ridge: 0.6, Seed points for topology preserving skeletonisation: false, Show Flinn plots: false; Show algorithm convergence, false; Show verbose output images, false*, to calculate Tb.EF
12. Run *Plugins > BoneJ > Purify* to remove small background particles
13. Run *Plugins > BoneJ > Connectivity (Modern)* to calculate Conn.D

### Scripting use case

Use the macro recorded from the steps above, edited to the below, taking care to place each run() command on a single line without any carriage returns. It is also possible to incorporate BoneJ plugins in Python scripts.


//Ensure that umzc_378p_Apteryx_haastii_head.tif is open and selected

//smooth the image
run("Gaussian Blur 3D...", "x=2 y=2 z=2");

//do the thresholding
setThreshold(26892, 65535);
setOption("BlackBackground", true);
run("Convert to Mask", "method=Default background=Dark");

//Calculate DA - remove the line wrapping in your macro code
run("Anisotropy", "directions=2000 lines=10000 samplingincrement=1.73
    recommendedmin=true printradii=false printeigens=false displaymilvectors=false");

//Calculate BV/TV
run("Area/Volume fraction");

//Calculate Tb.Th and Tb.Sp and display thickness maps
run("Thickness", "mapchoice=Both showmaps=true maskartefacts=true");

//Calculate EF - remove the line wrapping in your macro code
selectWindow("umzc_378p_Apteryx_haastii_head.tif");
run("Ellipsoid Factor", "nvectors=100 vectorincrement=0.435 skipratio=10
    contactsensitivity=1 maxiterations=50 maxdrift=1.73 runs=3 weightedaveragen=3
    seedondistanceridge=false distancethreshold=0.6 seedontopologypreserving=true
    showflinnplots=true showconvergence=true showsecondaryimages=false");

//Calculate Conn.D
selectWindow("umzc_378p_Apteryx_haastii_head.tif");
run("Purify", " ");
run("Connectivity (Modern)");


### Use case results

**Figure 2.**
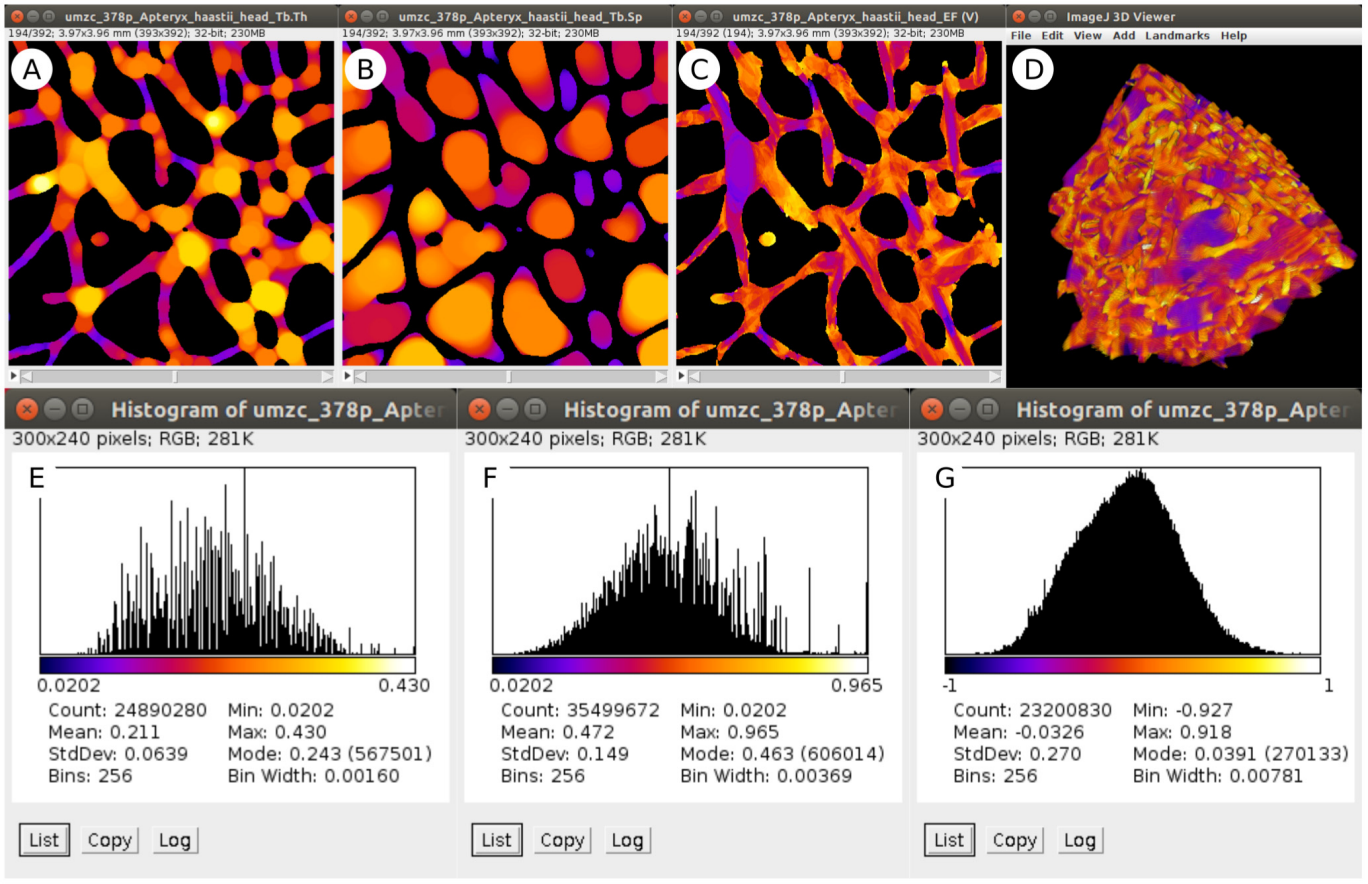
Sample image output from the worked example above. Stacks displaying maps of Tb.Th ( **A**), Tb.Sp ( **B**), Tb.EF ( **C**) contain pixels whose values represent the local thickness, separation or ellipsoid factor respectively, each with the *Fire* lookup table (LUT) applied and spread between the minimum and maximum pixel value. Background pixels are set to *NaN* (not a number) to exclude them from numerical analysis. Plotting histograms ( *Analyze > Histogram...*) of the output image stacks results in distributions and summary statistics for Tb.Th ( **E**), Tb.Sp ( **F**) and Tb.EF ( **G**): note the relation between the LUT and histogram x-axis values. Converting the EF image ( **C**) to RGB ( *Image > Type > RGB*) and loading in the 3D Viewer ( *Plugins > 3D Viewer*) results in an interactive 3D visualisation ( **D**).

Both interactive and scripted examples log numerical results to the BoneJ Result table ( Table 2) and produce output images that may be used for further analysis and to visualise the spatial distribution of measurements ( Figure 2).

**Table 2.** Expected results logged in the BoneJ result table comparing BoneJ2 and BoneJ1 output. Due to the stochastic nature of sampling both degree of anisotropy (DA) and *ellipsoid* factor (EF) vary between runs in an image- and setting-specific manner. Modern *Anisotropy* and *Ellipsoid Factor* ^16^ plugins differ in implementation from those in BoneJ1, so some minor variation in output is expected. Users are strongly encouraged to run sensitivity analyses. to select settings for *Anisotropy* and *Ellipsoid Factor* (as we have done) ^16^ that produce a stable result on their images. Note that *Thickness, Volume Fraction* and *Connectivity* are direct ports from BoneJ1 to BoneJ2 and have no stochastic character, so their results are expected to be invariant for the same input image. BoneJ2’s *Purify* uses *Particle Analyser’s* improved ConnectedComponents code ^19^ and completes in ~ 1 s compared to ~ 15 s in BoneJ1.

Image	BoneJ2 ( *styloid-r11*)	BoneJ 1.4.3
DA	0.51675	0.56665
**BV (mm ^3^)**	25.92792	25.92792
**TV (mm ^3^)**	62.21983	62.21983
**BV/TV**	0.41671	0.41671
**Tb.Th Mean (mm)**	0.21246	0.21246
**Tb.Th Std. Dev. (mm)**	0.06347	0.06347
**Tb.Th Max (mm)**	0.43277	0.43277
**Tb.Sp Mean (mm)**	0.47014	0.47014
**Tb.Sp Std. Dev. (mm)**	0.14888	0.14888
**Tb.Sp Max (mm)**	0.96242	0.96242
**Median EF**	-0.14632	NaN (mean, -0.21451)
**Max EF**	0.91779	0.91695
**Min EF**	-0.84867	-0.93064
**Euler char (χ)**	-291	-291
**Corr. Euler (χ – Δχ)**	-238.375	-238.375
**Connectivity**	239.375	239.375
**Conn.D (mm ^3^)**	3.84725	3.84725

### Developer use case

New BoneJ developers are encouraged to read the extensive developer documentation at BoneJ’s GitHub wiki. New developers are welcome to fork the repository, make and commit changes to their personal fork, and then submit a *pull request* (PR) to bonej-org/BoneJ2 for review and merging by the BoneJ team.

## Discussion

BoneJ2 made its first general release (semantic version 7.0.0, codenamed *styloid*) via the ImageJ updater in March 2020, and events with Modern versions began appearing with greater frequency in the usage report, with 1,000 – 4,000 events recorded weekly from 190 cities in 36 countries between March and June 2020.

### Limitations

During the experimental release phase, we noticed that naïve direct ports of BoneJ1 code to BoneJ2 using ImgLib2 could result in significant performance degradation, despite Rueden *et al.*’s claims of minimal performance loss or even performance enhancement using an Ops design ^8^. By instrumentation with timer code, and inspection of hotspots with a profiler (VisualVM, Oracle), we found that use of ImgLib2’s RandomAccess, setPosition() and get() methods can be very costly in computation resources compared to accessing pixel values from primitive arrays. Even after implementing multithreading and avoiding RandomAccess with a sequential pixel access design, Modern Connectivity (which uses a cursor) is about half the speed of Legacy Connectivity, which uses primitive array access to read pixel values. The conscious design choice to make pixel array access difficult with ImgLib2’s libraries to nudge developers to handle *N*-dimensional images can have the unfortunate side effect of making pixel access slow. Future BoneJ developments will balance the potential for generalisation of algorithms into *N*-dimensional images versus execution performance. Algorithms that function exclusively in 3 spatial dimensions will continue to use higher performance primitive array access rather than the slower *N*-dimensional approach of ImgLib2.

We were also held back by the realisation of new core ImageJ functionality lagging the proposals of the functionality by several years. We intended to incorporate sophisticated region of interest (ROI) handling in BoneJ2, but libraries to do so were not developed by the ImageJ team. In that regard, we have adopted a less ambitious practice of developing against currently available technologies and integrating new third-party technologies only after they are released to users. In retrospect we could have released the first experimental version earlier to make it easier for users to adopt the new software.

 The multi-module strategy allowed earlier releases and feedback but made the software more complex to manage because legacy code needs to be kept separate from modern code, which creates some duplicate work in the construction and distribution of BoneJ. The separation of concerns in BoneJ2’s engineering is intended to allow efficient adoption of new functionality but spreading the implementation across the ImageJ software stack has made the design of BoneJ more complicated. We must compile and build multiple components, and ensure they work together, before we can safely release a BoneJ version. We must also ensure that the changes we make to ImageJ (or other external code) are accepted, merged, and released before we can make the latest BoneJ available. On occasion we have had to revise our code that was already accepted into ImageJ to implement a detail that a BoneJ wrapper plugin required.

### Future

BoneJ will continue to be maintained and developed as opportunities arise to improve research tools.

### Funding

BoneJ’s development was initially supported as an unanticipated outcome of a BBSRC project grant (BB/F001169/1). The strategic value of software resources was recognised by MD while working as a postdoc, leading to the publication of the software and its paper and development of the initial project resources. Subsequent maintenance was performed ad-hoc as a service to the community alongside other job responsibilities but was not directly funded until a successful bid to Wellcome Trust (108442/Z/15/Z), after a similar bid to BBSRC was declined. BBSRC subsequently funded some development on BoneJ’s *Ellipsoid Factor* that was described as part of an ordinary project grant (BB/P006167/1).

In November 2019, in recognition of the need for specific funding for software and other technology development for the biosciences, Wellcome Trust split the Biomedical Resource and Technology Development Grant scheme that funded BoneJ2 into two parts, ringfencing funds for Technology Development Grants to "substantially enhance an existing technology or resource" ^20^. Ongoing maintenance of existing resources including the day-to-day servicing of user requests would appear not to satisfy the condition of "substantial enhancement" which could contribute to newly upgraded tools being abandoned due to lack of dedicated support personnel.

 Larger institutions (such as AAF’s current employer UCL) may decide to set up a core facility with central funds and/or accounting processes to recoup costs from researchers’ grants, to support research software engineers who service members of the institution’s research community. Embedding RSEs within individual research groups remains challenging due to RSEs needing specialist skills including research experience, yet funding schemes often require hiring a technician or postdoctoral scientist which are roles distinct from RSE. Institutions may also lack a suitable career structure that encapsulates RSEs’ pay, role, responsibilities, and expected output as service staff, rather than research staff ^21^. Professional Research Software Engineering societies have been established in (at least) the UK, US, Netherlands, Germany, and the Nordic countries, while the UK’s Software Sustainability Institute and UKRI e-infrastructure roadmap are actively highlighting these weaknesses in the funding and career landscape for individuals who may be interested in pursuing an RSE, rather than classical academic, career pathway. Given limited personnel funds available in many grant schemes, principal investigators may prefer to recruit an ‘all-rounder’ postdoctoral researcher who can code in addition to performing research. Our experience as a small project team with a PI (MD), RSE (RD), and PhD student then postdoc (AAF) highlighted the added value that a person with an engineering and service orientation brings to a scientific team. The BoneJ2 project also demonstrated the labour intensiveness of quality engineering practice which cannot be expected of a postdoctoral researcher, who is judged mainly on their academic writing output. The RSE is responsible for setting up and maintaining development practices that lead to more robust and reliable programs and user experience, which is vital when software is disseminated to other users, and when the project receives contributions from third parties. The RSE insists on good development practices, so that scientists’ new approaches to problems can be packaged in a manner suitable for public consumption, and in doing so enhance the impact and societal value of research.

## Summary

How functionality is achieved on the development side is a secondary concern for users, whose primary concern is getting results from data. Poor software development practice may not affect users’ experience of the software, but fast and undisciplined coding is likely to lead to more bugs and harder to solve errors. In the long run it may also become increasingly difficult to add new features and respond to changes. On the other hand, researchers are usually under time pressure to produce scientific results with little reason to dedicate the effort required to write sustainable software. An important part of writing maintainable code is to make it easy to read and understand. We posit that code clarity is especially important for research software, because researchers read program code to understand and adapt the methodology of an experiment, making code an essential part of the scientific record. A counterintuitive outcome of working on community software like BoneJ is that the more useful contributions are removed from the project and donated to the common pool upstream, which creates a need for developers to let go of ownership. Retaining code ownership for reasons of maintaining personal key performance indicators may be counterproductive to generating lasting impact from development work. BoneJ remains an open-source project with all changes and much of the discussion about its development occurring in the public domain; we consider process transparency an essential feature of scientific discourse.

## Data availability

All data underlying the results are available as part of the article and no additional source data are required.

## Software availability

1. Software available from: https://bonej.org/ and https://imagej.github.io/plugins/bonej
2. Source code available from: https://github.com/bonej-org/BoneJ2
3. Archived source code at time of publication: https://doi.org/10.5281/zenodo.1427262 ^18^
4. Licence: Simplified BSD-2 licence

## Notes

^i^ https://scholar.google.com/scholar?cites=16814410598979768336&scisbd=1
