# Supplementary material for: BoneJ2 - refactoring established research software
Source: Wellcome Open Res. 2021 Apr 28;6:37. Originally published 2021 Feb 22. [Version 2] doi: 10.12688/wellcomeopenres.16619.2 (PMC8063517; doi:10.12688/wellcomeopenres.16619.2)
Supplement: Peer review history

# Peer review: Reviewer response for version 2

**DOI:** 10.21956/wellcomeopenres.18585.r43626

**Published:** 2021-05-04

**Authors:** Kamel Madi, Loic Courtois

**Affiliations:** 1 3Dmagination Ltd, Didcot, UK

The authors have answered all my comments.

Are the conclusions about the tool and its performance adequately supported by the findings presented in the article?

Yes

Is the rationale for developing the new software tool clearly explained?

Yes

Is the description of the software tool technically sound?

Yes

Are sufficient details of the code, methods and analysis (if applicable) provided to allow replication of the software development and its use by others?

Yes

Is sufficient information provided to allow interpretation of the expected output datasets and any results generated using the tool?

Yes

Reviewer Expertise:

X-ray computed tomography, advanced image processing, plugins development.

We confirm that we have read this submission and believe that we have an appropriate level of expertise to confirm that it is of an acceptable scientific standard.

# Peer review: Reviewer response for version 2

**DOI:** 10.21956/wellcomeopenres.18585.r43625

**Published:** 2021-04-28

**Authors:** Mariana Kersh

**Affiliations:** 1 Mechanical Science and Engineering, University of Illinois at Urbana-Champaign, Urbana, IL, USA; 2 Beckman Institute for Advanced Science and Engineering, University of Illinois at Urbana-Champaign, Urbana, IL, USA

I think the inclusion of the previous version outputs to the current BoneJ2 output data helps to demonstrate that the implementation of the new structure for BoneJ2 has been done properly/doesn't result in significant changes. This will certainly give the community confidence when making the transition to BoneJ2.

 Thank you again for your contributions.

Are the conclusions about the tool and its performance adequately supported by the findings presented in the article?

Partly

Is the rationale for developing the new software tool clearly explained?

Yes

Is the description of the software tool technically sound?

Yes

Are sufficient details of the code, methods and analysis (if applicable) provided to allow replication of the software development and its use by others?

Yes

Is sufficient information provided to allow interpretation of the expected output datasets and any results generated using the tool?

Partly

Reviewer Expertise:

Musculoskeletal biomechanics. Structure-function in bone and passive tissues.

I confirm that I have read this submission and believe that I have an appropriate level of expertise to confirm that it is of an acceptable scientific standard.

# Peer review: Reviewer response for version 1

**DOI:** 10.21956/wellcomeopenres.18322.r42731

**Published:** 2021-04-21

**Authors:** Deborah Schmidt

**Affiliations:** 1 Max Delbrück Center for Molecular Medicine in the Helmholtz Association, Bonn, Germany

The publication BoneJ2 - refactoring established research software captures the effort of maintaining and modernizing research software while adjusting to infrastructural changes in the community and ensuring backwards compatibility. The authors depict in detail the longstanding process of moving the ImageJ plugin suite for skeletal biology image analysis first released in 2010 into the ImageJ2 / SciJava ecosystem. The approach described in the paper to refactor the existing code base in an organized fashion can serve as a blueprint for other projects to follow their footsteps, i.e. by clearly separating plugins into legacy and modern components, a high percentage of unit test coverage, and analyzing and documenting analysis divergences after refactoring plugins.

 The design was adjusted to the modularity of SciJava by creating independent, reusable pieces and I highly commend the efforts made to move general purpose code into the ImageJ2 / SciJava core libraries such as imagej-ops and SCIFIO. Both SCIFIO as well as ImgLib2 deserve to be referenced though.

 The paper is educating for research groups maintaining software, mentioning the challenges of the ecosystem and the amount of work that isn't achievable for a scientist in part-time, but was executed by a dedicated Research Software Engineer. Same applies to the depicted funding challenges like changes to the Wellcome Trust seemingly hindering the ongoing financial support of maintaining BoneJ2.

 Strategies to ease the user into switching to new implementations of plugins are mentioned. It would be valuable to read about any feedback the authors might have received from their users regarding the refactoring process. Since the usage of opt-in Google analytics was mentioned, insightful statistics could be included.

Are the conclusions about the tool and its performance adequately supported by the findings presented in the article?

Yes

Is the rationale for developing the new software tool clearly explained?

Yes

Is the description of the software tool technically sound?

Yes

Are sufficient details of the code, methods and analysis (if applicable) provided to allow replication of the software development and its use by others?

Yes

Is sufficient information provided to allow interpretation of the expected output datasets and any results generated using the tool?

Yes

Reviewer Expertise:

Software engineering, molecular biology.

I confirm that I have read this submission and believe that I have an appropriate level of expertise to confirm that it is of an acceptable scientific standard.

# Peer review: Reviewer response for version 1

**DOI:** 10.21956/wellcomeopenres.18322.r42735

**Published:** 2021-04-13

**Authors:** Mariana Kersh

**Affiliations:** 1 Mechanical Science and Engineering, University of Illinois at Urbana-Champaign, Urbana, IL, USA; 2 Beckman Institute for Advanced Science and Engineering, University of Illinois at Urbana-Champaign, Urbana, IL, USA

This paper describes the rationale and methods used to update BoneJ – an open-source plug-in of ImageJ which is widely used within the bone community (as well as other research areas making use of morphometric analyses of structures). One motivation for this work was the update of ImageJ to ImageJ2 which resulted in third party libraries, which BoneJ relied on, being deleted or no longer maintained.

 The description of these changes and the subsequent enormous amount of work involved in adapting to this is well described at a high-level and very much appreciated. The call for more transparency in science in general includes software methods. I congratulate the authors on their contributions towards this cause and applaud their call to attention to the dilemma they (and others) face that choose to contribute to an effort that is needed by the community but usually not recognized for advancement career-wise. This is something our community needs to address and is an important component of this paper.

 With respect to their development efforts for BoneJ2, my background is not in software development, so I write this review as an “informed user”.

 If the first purpose of this paper is to the updates to BoneJ2 that ensure compatibility with ImageJ2/Fiji, it would be helpful to see a few side-by-side examples or other performance metrics. For example, in the use case shown– it’s not clear to me how I should interpret these results (Table 2, Figure 2). What is the “expected” result? Would it make sense to compare this to the legacy structure to demonstrate that the modernized structure of the plug-in still functions properly and/or with similar processing times, etc. I understand that the way the data is sampled for some of the metrics may result in some differences, but overall, the values should still be quite close. A second option to demonstrate robustness would be to report the standard deviations (or range) of results between repeated runs on the same dataset.

 Thanks again to the developers for their contributions to an important tool. I hope others, including funding agencies and senior academics that evaluate career progress, recognize the value of such contributions.

Are the conclusions about the tool and its performance adequately supported by the findings presented in the article?

Partly

Is the rationale for developing the new software tool clearly explained?

Yes

Is the description of the software tool technically sound?

Yes

Are sufficient details of the code, methods and analysis (if applicable) provided to allow replication of the software development and its use by others?

Yes

Is sufficient information provided to allow interpretation of the expected output datasets and any results generated using the tool?

Partly

Reviewer Expertise:

Musculoskeletal biomechanics. Structure-function in bone and passive tissues.

I confirm that I have read this submission and believe that I have an appropriate level of expertise to confirm that it is of an acceptable scientific standard, however I have significant reservations, as outlined above.

# Response

**Published:** 2021-04-21

**Authors:** Michael Doube

**Affiliations:** City University of Hong Kong, Hong Kong

We thank the reviewer for their understanding and support of this manuscript.

*If the first purpose of this paper is to the updates to BoneJ2 that ensure compatibility with ImageJ2/Fiji, it would be helpful to see a few side-by-side examples or other performance metrics. For example, in the use case shown– it’s not clear to me how I should interpret these results (Table 2, Figure 2). What is the “expected” result? Would it make sense to compare this to the legacy structure to demonstrate that the modernized structure of the plug-in still functions properly and/or with similar processing times, etc. I understand that the way the data is sampled for some of the metrics may result in some differences, but overall, the values should still be quite close. A second option to demonstrate robustness would be to report the standard deviations (or range) of results between repeated runs on the same dataset.*

 We have added a column to table 2 that includes the results from BoneJ 1.4.3 (the final release of BoneJ1) after running the same interactive use case, showing that the non-stochastic plugins produce identical results in legacy and modern implementations.

 The stochastic plugins that rely on random sampling *Anisotropy* and *Ellipsoid Factor* have had some improvements made to their implementations between BoneJ1 and BoneJ2 so we don't expect a precise reproduction of DA and EF values, however, they should be close.

 We add some description of Table 2 to the text to aid the reader in their interpretation of the results and some further description of e.g. improvements to *Purify*'s execution speed. We also direct the reader to a new publication on *Ellipsoid Factor* in which thorough sensitivity analyses were performed, and encourage readers to do sensitivity analysis (running the analysis repeatedly with a range of reasonable input settings and observing the variation in output) as a matter of course when developing their image processing pipelines.

 It is important to emphasise that in testing and for validation of correct function, simple test images with well-known properties are used to ensure that the new code isn't simply repeating the mistakes of old code to get the same result. Instead both sets of code must independently calculate the correct (expected) result from the same image. The example image provided for the use case is too complicated to use for validation in a meaningful way because it is not obvious, *a priori*, what the precise results should be. It is, however, useful for demonstrating real-world performance in this comparison of old and new code.

# Peer review: Reviewer response for version 1

**DOI:** 10.21956/wellcomeopenres.18322.r42734

**Published:** 2021-03-18

**Authors:** Kamel Madi, Loic Courtois

**Affiliations:** 1 3Dmagination Ltd, Didcot, UK

The article raises an important concern about BoneJ2 longevity and open source software in general. The key challenges and technical steps taken to improve the quality of BoneJ code are well described in the introduction. The efforts made by the authors and the process of transparency are greatly appreciated due to the difficult task undertaken to make BoneJ2 a robust tool. The progressive transition from legacy to modern architecture is nicely detailed; the description is very technical though (Design/Implementation section) which is fine for an audience like developers or RSE but could be difficult to comprehend from a "user" point of view. From a user point of view, (i) the interactive use case and the expected results are very useful to test the plugin and validate the results, (ii) the limitations of the new implementation, and in particular accessing pixel values, will be useful information to the community during the transition period. The authors have done a great job at raising awareness on the importance of funding, training and crucial role played by permanent staff such as RSE to maintain the code. The paper could help setting examples that others could follow and help growing concern and giving more resources to teams working on open source packages such as BoneJ.

 Comments/suggestions:

1. Interactive use case: step 11 did not work for me. After running the ellipsoid factor plugin, looks like something is running but never ends. 
2. Could be interesting to validate the results provided in the use case against another software that uses a similar method (focus on just trabecular thickness for example).

Are the conclusions about the tool and its performance adequately supported by the findings presented in the article?

Yes

Is the rationale for developing the new software tool clearly explained?

Yes

Is the description of the software tool technically sound?

Yes

Are sufficient details of the code, methods and analysis (if applicable) provided to allow replication of the software development and its use by others?

Yes

Is sufficient information provided to allow interpretation of the expected output datasets and any results generated using the tool?

Yes

Reviewer Expertise:

X-ray computed tomography, advanced image processing, plugins development.

We confirm that we have read this submission and believe that we have an appropriate level of expertise to confirm that it is of an acceptable scientific standard.

# Response

**Published:** 2021-04-19

**Authors:** Michael Doube

**Affiliations:** City University of Hong Kong, Hong Kong

Thank you for the comments and support.

*1. Interactive use case: step 11 did not work for me. After running the ellipsoid factor plugin, looks like something is running but never ends.*

 There is a typo in the *Vectors* setting, which should be 100, not 1, and is fixed in the resubmission. Each ellipsoid is represented as a set of unit vectors multiplied by the ellipsoid's 3×3 matrix. Collisions between the ellipsoid and the foreground/background boundary are calculated for each of the unit vectors to determine whether or not it is fully contained within foreground. So while it is not strictly illegal to use only 1 vector, the sampling is too sparse to have much meaning. We have found that 100 is a reasonable compromise between sampling density and efficiency, and believe there are more efficient sampling methodologies to discover than the one employed so far.

*2. Could be interesting to validate the results provided in the use case against another software that uses a similar method (focus on just trabecular thickness for example).*

 Comparisons with other software have been performed before on BoneJ's algorithms (e.g. EF vs SMI vs SMI in https://doi.org/10.3389/fendo.2015.00162) but are not really validation. Validation requires that a ground truth or expected result is known. In 'validating' against other software, one is making an assumption that the other software has no faults and makes a similar representation of the data as the software under test. It is possible that both sets of software get the same wrong answer, for example, or get the same answer by chance rather than design. If they get different answers there is no way to tell which (if any) of them got the right answer without reference to ground truth. If the other software has been validated against ground truth, then there is no advantage to testing against the other software rather than against ground truth directly. The primary value in software comparisons is to expose biases produced by different implementations of an algorithm when they are run on the same data, or to explore the effects of different settings such as pixel size:feature size or image pre-filtering. The possibilities for these comparisons are many and domain specific, and as such are outside the scope of this manuscript.

 Algorithms in BoneJ are validated internally by unit tests on synthetic data for which expected results are readily calculated, and by integration tests that ensure that results on real test images are stable.
